# Supplementary material for: Metasurface-assisted phase-matching-free second harmonic generation in lithium niobate waveguides
Source: Nat Commun. 2017 Dec 13;8:2098. doi: 10.1038/s41467-017-02189-6 (PMC5727391; doi:10.1038/s41467-017-02189-6)
Supplement: Supplementary file 1 — Supplementary Information [file 41467_2017_2189_MOESM1_ESM.pdf]

Supplementary Note 1 | Mode conversion after a single set of phased antennas

We conducted simulations to study mode conversion by a single amorphous silicon (a-Si) phased antenna array at λ = 775 nm. In the simulations, the fundamental TE_{00} mode is launched into the input port of a lithium niobite ($LiNbO_3$) waveguide. Supplementary Figure 1 shows that the output is a mixture of higher-order modes as a result of the interaction between the TE_{00} mode and the phased antenna array. Our modal analyses show that 39% of the output power is carried by the TE_{20} mode, 7% by the TE_{60} mode, 5% by the TM_{30} mode and 11% by a combination of other higher-order modes, and that 38% of the output power is still carried by the fundamental TE_{00} mode (unconverted).

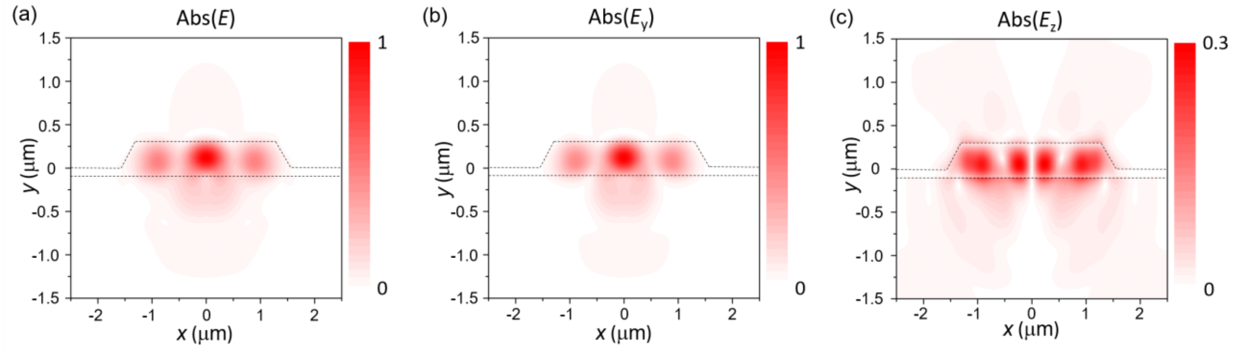

Supplementary Figure 1 | Mode profile after a single phased antenna array. The electric field amplitude (a), E_y (b) and E_z (c) profile after the TE_{00} mode at λ = 775 nm interacts with one antenna array.

Supplementary Note 2 | Phased-antenna design and simulation techniques

In Lumerical FDTD simulations, the $LiNbO_3$ waveguide is orientated along the z direction, and the cross section of the waveguide is in the **x-y** plane. The optical refractive indices of $LiNbO_3$ are obtained from the Palik database[1] and are anisotropic with n_o (ordinary index of refraction) in the **y** and **z** directions and n_e (extraordinary index of refraction) in the **x** direction. n_e used in FDTD simulations is shown in Supplementary Figure 2c. The diagonal terms of the second-order nonlinear susceptibility tensor of $LiNbO_3$ are set to be $\chi^{(2)}_{xxx}$ = 66 pm/V, $\chi^{(2)}_{zzz}$ = 6 pm/V and $\chi^{(2)}_{yyy}$ = 0 pm/V. The optical refractive indices of a-Si are obtained from ellipsometric measurements and are shown in Supplementary Figure 2b). The optical refractive indices of SiO_2 are also from the Palik database. The amplitude of incident fundamental waveguide mode is set to be 10^9 V/m.

The phase response of a-Si nano-antennas is obtained also using FDTD simulations. The width and height of the nano-antennas are kept constant (75 nm). The centre-to-centre distance between adjacent antennas is kept to be dz = 140 nm. These numbers are chosen according to our fabrication capabilities. In our FDTD simulations, a nano-antenna is placed on a $LiNbO_3$ substrate and oriented along the **x** axis as shown in the inset of Supplementary Figure 2a. The extraordinary index of refraction, n_e, of $LiNbO_3$ is along the **x** axis. An x-polarized plane wave at λ = 775 nm is incident on the nano-antenna from the substrate, and the phase of scattered light from the nano-antenna is monitored as a function of the length of the antenna (Supplementary Figure 2a). The scattering phase monotonically increases as a function of the length of the nano-antennas. The gradient metasurface is created by assembling an array of such nano-antennas with the phase difference between adjacent elements to be 0.5 degrees.

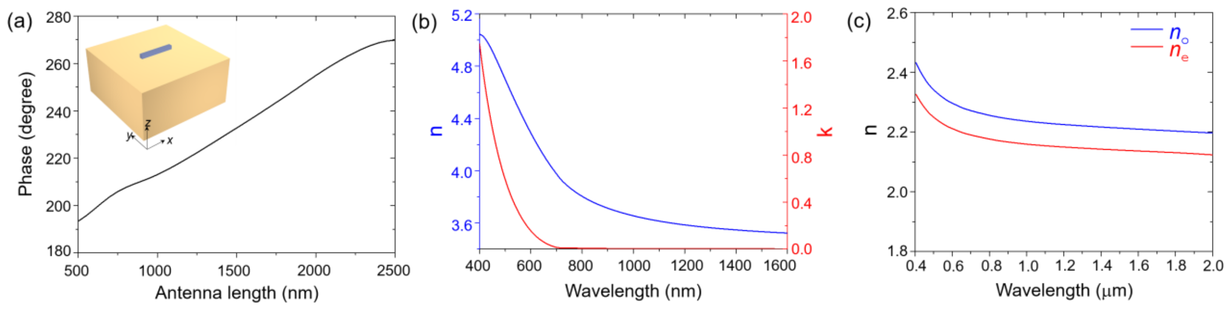

Supplementary Figure 2 | Phased-antenna design parameters. (a) Phase of scattered light waves from amorphous silicon nano-antennas patterned on a $LiNbO_3$ substrate as a function of nano-antenna length. The inset shows the configuration used in FDTD simulations. The antenna rod is along the **x** axis. A plane wave at λ = 775 nm with **x** polarization is incident on the antenna from the substrate. (b) Complex optical refractive indices of amorphous silicon, measured using ellipsometry and used in FDTD simulations. (c) Extraordinary index of refraction of $LiNbO_3$ used in FDTD simulations. The extinction coefficient is assumed to be zero.

Supplementary Note 3 | Mode power loss through the antenna region

One major advantage of using dielectric a-Si nano-antennas is that the insertion loss is greatly reduced compared to plasmonic nano-antennas. We conducted simulations to study the propagation loss of five different modes (i.e., TE_{00}, TE_{20}, TE_{40}, TM_{30} and TM_{40}) as a result of their interaction with a single phased antenna array at three different wavelengths: λ = 750 nm, 775 nm and 800 nm (Supplementary Figure 3). This propagation loss is due to undesired optical scattering by the antennas into air or the substrate, as well as power absorption by the antennas. The simulation results show that for the TE_{00} mode, the power loss is 7%, 12% and 14% of the input power at λ = 750 nm, 775 nm and 800 nm, respectively. Similarly, the power loss is 10%, 12%, and 9% for the TE_{20} mode, and 11%, 11% and 9% for the TE_{40} mode at the three wavelengths,

respectively. As for the TM modes, the propagation loss is much smaller. For example, the power loss is 1.7%, 1.6% and 1.6% for the TM_{30} mode, and 2.1%, 2.0% and 2.5% for the TM_{50} mode at those three wavelengths, respectively. Overall, the insertion losses are small in our all-dielectric devices.

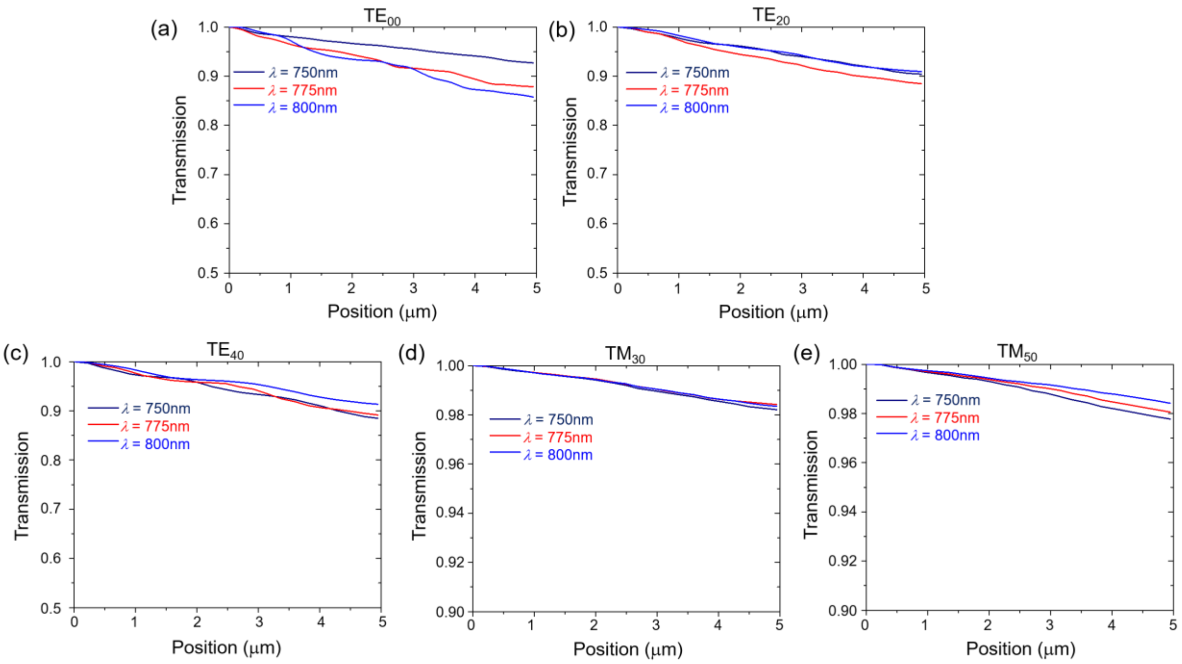

Supplementary Figure 3 | Optical power loss after a single phased antenna array. Optical power maintained inside the $LiNbO_3$ waveguide as a function of propagation distance when different waveguide modes [TE_{00} (a), TE_{20} (b), TE_{40} (c), TM_{30} (d) and TM_{50} (e)] at three different second harmonic frequencies interact with a single phased antenna array.

Supplementary Note 4 | Power evolution after phased antenna arrays

To investigate how the generated SH signal evolves after the phased antenna arrays, we conducted full-wave simulations to monitor the SHG power at λ = 775 nm up to a propagation distance of 100 μm for three different devices, which contain one, three and five phased antenna arrays, respectively. The simulation results show that the SHG power monotonically increases in the antenna-array-covered waveguide section (red curves in Supplementary Figure 4) and then oscillates with a small amplitude in the following bare waveguide (black curves in Supplementary Figure 4).

As stated in the main text, in the antenna-array-covered section, the fundamental mode at λ ~ 775 nm unidirectionally couples into higher-order modes. As a result, optical power is continuously transferred from the pump at λ = 1550 nm to the SH signal, leading to a monotonic increase of the SHG power. After the antenna-array-covered section, there is no further mode conversion at the SH wavelength. Each converted higher-order mode at λ ~ 775 nm interacts with the fundamental mode at λ = 1550 nm weakly and thus optical power is coupled back and forth between the two modes in a period way determined by their phase mismatch. This effect could lead to a periodic variation of the SHG power. In addition, different higher-order modes at λ ~ 775 nm propagate with different phase velocities and their interference could also cause variations of the SHG power along the waveguide. In summary, the SHG power is accumulated monotonically in the antenna-array-covered section and maintains its intensity (although with small oscillations) after that section.

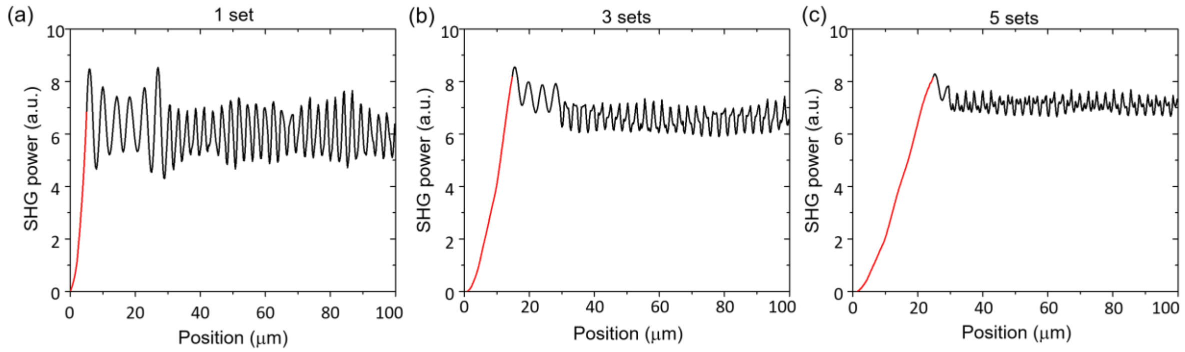

Supplementary Figure 4 | Power evolution after phased antenna arrays. Second harmonic power as a function of propagation distance in three devices patterned with 1 (a), 3 (b) and 5 (c) sets of phased antenna arrays. Second harmonic power increases monotonically in the waveguide section patterned with the antenna arrays (red) but oscillates in the following bare waveguide section.

Supplementary Note 5 | Robustness against the waveguide parameters

We conducted full-wave simulations to study the effects of waveguide geometries, such as waveguide width and etching thickness, on device performance (Supplementary Figure 5). The simulation results show that the performance is tolerant to the waveguide geometry changes. The change to the SHG power in a device with three sets of phased antenna arrays is small when the lengths of the nano-rod antennas deviate from their designed values by ± 10% (a), when the antenna arrays are offset from the centre of the waveguide up to 100 nm (b), when the width of the $LiNbO_3$ waveguide deviates from its designed values by ± 100 nm (c), and when the waveguide etching depth deviates from its designed value by ± 50 nm (d). For example, Supplementary Figure 5c compares the SHG enhancement for waveguides of different widths (i.e., 2600 nm as used in fabricated devices, 2500 nm and 2700 nm as controls) patterned with three phased antenna arrays. The SHG enhancement at the end of the antenna arrays for these three waveguides exhibits a

variation of ± 6%. Supplementary Figure 5d shows that the performance is robust against variations of the waveguide etching depth. For three different etching depths of 300 nm (as used in fabricated devices), 250 nm and 350 nm of thin-film $LiNbO_3$, the SHG enhancement factors exhibit a variation of ± 1.2%.

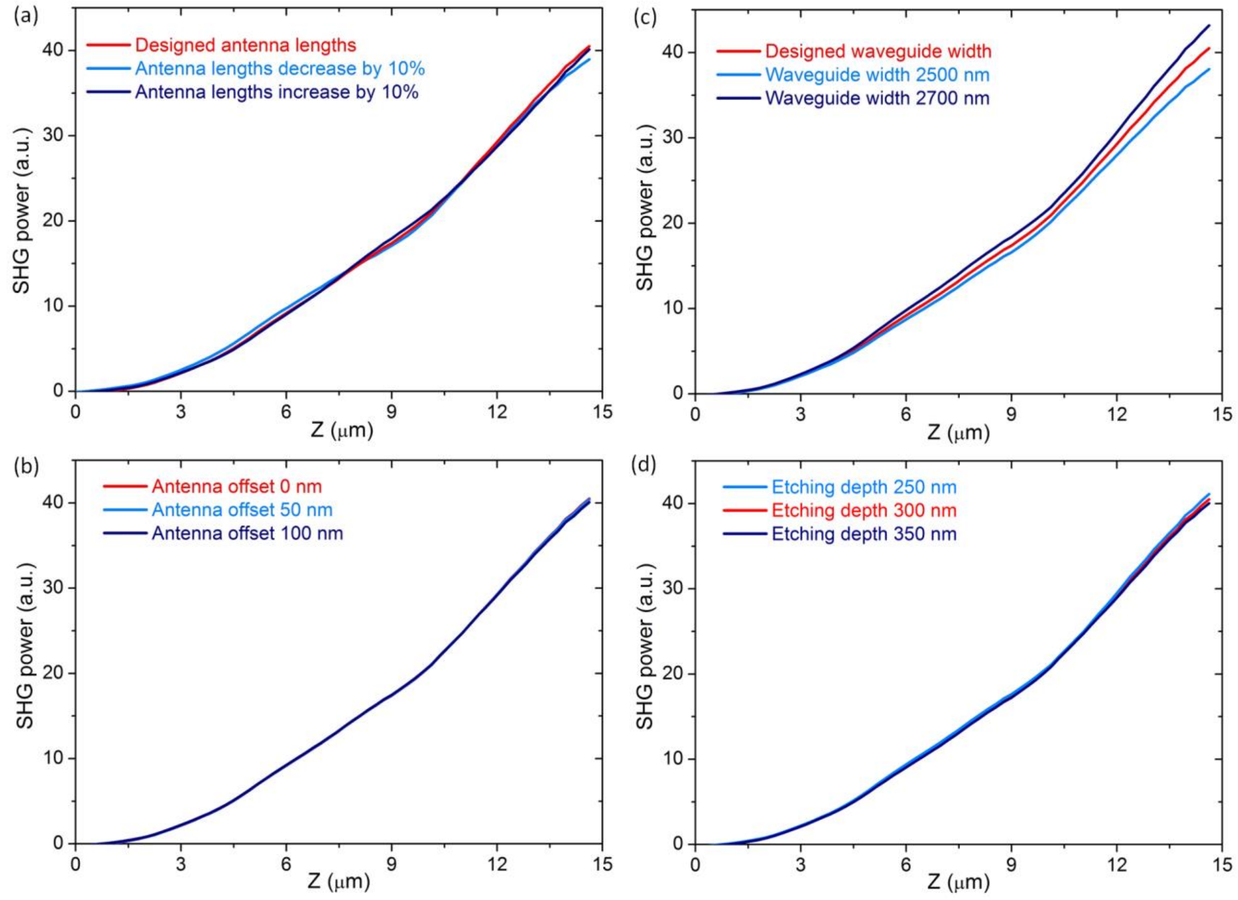

Supplementary Figure 5 | System robustness to fabrication variations. Simulations showing that with the new scheme of phase-matching-free nonlinear generation, the second harmonic generation process is insensitive to the variation of the geometric parameters of the devices, including the lengths of nano-rod antennas (a), the offset of the antenna arrays from the centre of the waveguide (b), the waveguide width (c) and the device etching depth (d).

Supplementary Note 6 | Second harmonic generation peak at 1667 nm

SH peaks are observed at λ ~ 1667 nm for all devices. They are caused by accidental phase matching between the $TE_{00}(\omega)$ mode and the $TE_{06}(2\omega)$ mode for our $LiNbO_3$ waveguide dimensions. Supplementary Figure 6 shows the simulated dispersion relations for different optical modes at both wavelengths in our $LiNbO_3$ waveguides. The modal index crossing near λ_{pump} ~ 1667 nm indicates accidental phase matching between the TE_{00} mode at the pump wavelength and the TE_{06} mode at the SH wavelength.

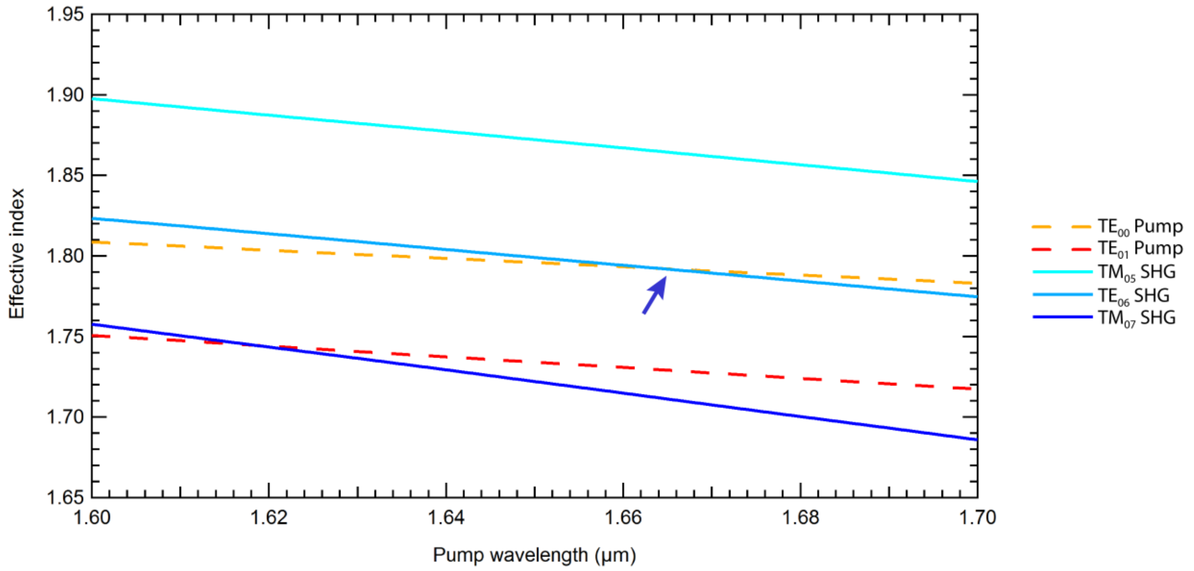

Supplementary Figure 6 | Accidental phase matching wavelength. Simulated modal effective index versus pump wavelength for different waveguide modes at both wavelengths. Arrow shows the accidental phase matching point at λ_{pump} ~ 1667 nm between TE_{00} mode at the pump wavelength and TE_{06} mode at the second harmonic wavelength.

Supplementary References

1. Palik, E. D. *Handbook of optical constants of solids.* Vol. 3 (Academic press, 1998).
